# Ferroptosis in COVID-19-related liver injury: A potential mechanism and therapeutic target

**DOI:** 10.3389/fcimb.2022.922511

**Published:** 2022-07-27

**Authors:** Yunqing Chen, Yan Xu, Kan Zhang, Liang Shen, Min Deng

**Affiliations:** ^1^ Department of Infectious Diseases, Affiliated Hospital of Jiaxing University, Jiaxing, China; ^2^ Department of Cardiology, Affiliated Hospital of Jiaxing University, Jiaxing, China

**Keywords:** COVID-19, ferroptosis, liver, SARS-CoV-2, hyperferritinemia

## Abstract

The outbreak and worldwide spread of coronavirus disease 2019 (COVID-19), which is caused by severe acute respiratory syndrome coronavirus 2 (SARS-CoV-2), has been a threat to global public health. SARS-CoV-2 infection not only impacts the respiratory system but also causes hepatic injury. Ferroptosis, a distinct iron-dependent form of non-apoptotic cell death, has been investigated in various pathological conditions, such as cancer, ischemia/reperfusion injury, and liver diseases. However, whether ferroptosis takes part in the pathophysiological process of COVID-19-related liver injury has not been evaluated yet. This review highlights the pathological changes in COVID-19-related liver injury and presents ferroptosis as a potential mechanism in the pathological process. Ferroptosis, as a therapeutic target for COVID-19-related liver injury, is also discussed. Discoveries in these areas will improve our understanding of strategies to prevent and treat hepatic injuries caused by COVID-19.

## Introduction

Since it was first reported in December 2019, severe acute respiratory syndrome coronavirus 2 (SARS-CoV-2) has become a severe threat to public health. The World Health Organization (WHO) declared the SARS-CoV-2 infection epidemic an international public health emergency, naming it coronavirus disease 2019 (COVID-19) on 11 February 2020. According to the WHO COVID-19 dashboard, the number of confirmed cases of COVID-19 has exceeded 500 million, leading to more than six million deaths.

SARS-CoV-2 is an RNA virus and has a tropism for cells expressing angiotensin-converting enzyme 2 (ACE2) receptors (Wan et al., 2020). Respiratory symptoms, such as fever, pharyngalgia, and dry cough, are the most common complaints from patients with COVID-19. However, the lung is not the only organ affected by SARS-CoV-2; the virus can affect various systems and result in multiple organ failure (Zaim et al., 2020).

Ferroptosis is a relatively novel cell death type that was first termed by Dixon et al. in 2012 (Dixon et al., 2012). Ferroptosis is an iron-dependent regulated cell death (RCD) characterized by iron overload and lipid peroxidation. The main morphological features of ferroptosis are mitochondrial shrinkage accompanied by increased mitochondrial membrane density and degenerated mitochondrial crista without changes in the nucleus (Dixon et al., 2012). Ferroptosis is regulated by several metabolic pathways, including iron, lipid, and amino acid metabolisms (Yan et al., 2021). Once the balance of iron absorption, storage, and exportation is disrupted, excessive cytosolic Fe^2+^ catalyzes the Fenton reaction and activates iron-dependent metabolic enzymes, leading to the production of highly reactive hydroxyl radicals (·OH) and oxidized polyunsaturated fatty acids (PUFAs) and eventually promoting the accumulation of lipid reactive oxygen species (ROS) and ferroptosis (He et al., 2022). In contrast, the metabolism of amino acids, especially the system Xc^–^glutathione–glutathione peroxidase 4 (system Xc^-^/GSH/GPX4) axis, is central to eliminating lipid ROS, with GPX4 regarded as a key regulator of ferroptosis (Yang et al., 2014). During ferroptosis, GPX4 expression is downregulated, while iron absorption and PUFA oxidation are upregulated. Additionally, two GPX4-independent pathways, nicotinamide adenine dinucleotide phosphate–ferroptosis suppressor protein 1–Coenzyme Q10 (Bersuker et al., 2019; Doll et al., 2019) and guanosine triphosphate cyclohydrolase 1–tetrahydrobiopterin/dihydrobiopterin axis (Kraft et al., 2020), allegedly participate in the regulation of ferroptosis.

At present, ferroptosis can be induced by four classes of compounds collectively called ferroptosis-inducing agents (FINs). These include (i) FINs that inhibit system Xc^-^ and prevent cystine imports, like erastin, sorafenib, and sulfasalazine; (ii) FINs that directly inhibit GPX4, such as Ras-selective lethal small molecule 3 (RSL3), (1S,3R)-RSL3; (iii) FINs that degrade GPX4, for example, FIN56; and (iv) FINs that indirectly inhibit GPX4 (Li et al., 2020).

Researchers from many fields have meticulously investigated ferroptosis in the past decade and have proposed it as a novel therapeutic target for a variety of diseases, including cancer (Alvarez et al., 2017; Lee et al., 2020), ischemia/reperfusion injury (Friedmann Angeli et al., 2014; Fang et al., 2019), and neurodegenerative disorders (Do Van et al., 2016; Park et al., 2021). Recently, the role of ferroptosis in various types of liver diseases, for instance, hepatitis, non-alcoholic fatty liver disease (NAFLD), liver cirrhosis, and hepatocellular carcinoma, has been explored as well (Chen et al., 2022). Liver injury has been reported as a common feature in COVID-19 (Amin, 2021); however, to date, no investigation has elucidated the potential role of ferroptosis in COVID-19-related liver injury. Recent studies showed that hepatitis virus caused liver injury through ferroptosis (Komissarov et al., 2021; Liu et al., 2021). In addition, chrysophanol could attenuate hepatitis B virus-induced hepatic fibrosis by inhibiting ferroptosis (Kuo et al., 2020). Hence, ferroptosis might also participate in SARS-CoV-2 infection-associated liver injury.

In this review, we deliberate current evidence pointing to a potential pathogenic role for ferroptosis in COVID-19-related liver injury and discuss potential therapeutic options with ferroptosis as the target.

## COVID-19 and liver injury

Although SARS-CoV-2 mostly affects the respiratory system, the virus also causes the dysfunction of other organs. A few studies have shown that liver injury occurs in patients with COVID-19 (Huang et al., 2020; Guan et al., 2020; Fan et al., 2020; Cai et al., 2020). COVID-19-related hepatic injury is characterized primarily by elevated levels of alanine aminotransferase and/or aspartate aminotransferase as well as macrovesicular and microvesicular steatosis, lobular and portal inflammation, ductular proliferation, and liver cell necrosis (Nardo et al., 2021). Additionally, decreased albumin and increased total bilirubin levels, along with alkaline phosphatase and gamma-glutamyl transferase, have been reported in COVID-19 patients (Wang et al., 2020; Yadav et al., 2021). However, not all SARS-CoV-2-infected patients have elevated transaminase. Patients with severe COVID-19 are more likely to have increased liver enzymes compared to non-severe COVID-19 patients (Guan et al., 2020). Furthermore, patients with viral hepatitis have a higher risk of severe COVID-19 (Hariyanto et al., 2022), and the risk could increase further in the presence of other comorbidities such as diabetes and cardiovascular diseases (Zhou et al., 2020; Gold et al., 2020). Moreover, NAFLD (Singh et al., 2021), cirrhosis (Choudhary et al., 2021; Qi et al., 2021), and hepatic carcinoma (Chagas et al., 2020) increase the risk of progression to severe COVID-19, which might be partly because the expression of ACE2 is significantly amplified in liver fibrotic/cirrhotic conditions (Paizis et al., 2005; Huang et al., 2009).

Recently, Sonzogni et al. found SARS-CoV-2 in liver samples from deceased COVID-19 patients (Sonzogni et al., 2020). In addition, Wanner *et al. (* Wanner et al., 2022) detected SARS-CoV-2 RNA in approximately 70% of autopsied liver specimens and isolated infectious SARS-CoV-2 from liver tissues postmortem. They also established that SARS-CoV-2 liver tropism is associated with the upregulation of interferon (IFN) responses, providing comprehensive evidence of the direct impact of SARS-CoV-2 on the liver.

A broad spectrum of potential mechanisms of COVID-19-associated hepatic dysfunctions has been proposed, including direct cytotoxicity due to the active viral replication of SARS-CoV-2 in hepatocytes and biliary epithelial cells, immune and cytokine-mediated liver destruction stemming from severe inflammatory responses, coagulopathy-driven vascular microthrombosis, endotheliopathy resulting from hypoxic and/or ischemic injury, congestion from right heart failure, and drug-induced liver injury (Amin, 2021; Nardo et al., 2021; Moreira et al., 2021).

## The link between COVID-19 and ferroptosis

Nowadays, several studies have provided evidence of ferroptosis in COVID-19. The ferroptosis signature was first reported in a 48-year-old male COVID-19 patient with cardiogenic shock (Jacobs et al., 2020). In this case, the authors found that E06 staining, which reflects lipid peroxidation during ferroptosis, was positive in the patient’s cardiomyocytes. Additionally, staining with E06 and 4-hydroxynonenal (a reactive breakdown product of lipid peroxides that carry out ferroptosis) was positive in renal proximal tubulin. However, it is unclear whether ferroptosis is directly induced by SARS-CoV-2 or if it is secondary to the pathological processes caused by COVID-19, such as ischemia/reperfusion injury. A Vero cell study found a significantly low mRNA expression of GPX4 after SARS-CoV-2 infection (Wang et al., 2021). In another investigation, the lung tissues of hamsters infected with SARS-CoV-2 exhibited increased apoptosis and ferroptosis (Bednash et al., 2022), indicating that SARS-CoV-2 possibly has a direct effect on ferroptosis. Most recently, Han et al. (Han et al., 2022) determined that SARS-CoV-2 infected human embryonic stem cell-derived SAN-like pacemaker cells and highlighted ferroptosis as a potential mechanism for cardiac arrhythmias in COVID-19 patients and deferoxamine (DFO, an iron chelator) as a drug candidate for blocking SARS-CoV-2 infection and subsequent ferroptosis.

In fact, many clues point to ferroptosis as participating in COVID-19. As the name implies, iron is pivotal to ferroptosis because erastin-induced cell death depends on iron rather than other metal ions (Dixon et al., 2012). Iron metabolism is one of the major pathways that trigger ferroptosis (Chen et al., 2020). As with human immunodeficiency virus, human cytomegalovirus, and hepatitis B virus, SARS-CoV-2 replication also requires iron; therefore, it is likely that more iron would be transferred into cells through transferrin receptor 1, activating the Fenton reaction and generating excessive lipid ROS. In addition, COVID-19 has recently been proposed as a part of the hyperferritinemic syndrome because of several similar clinical and laboratory characteristics, including high serum ferritin and cytokine storm (Perricone et al., 2020; Colafrancesco et al., 2020). The excess of ferritin could contribute to the accumulation of cellular iron through ferritinophagy, eventually resulting in ferroptosis (Mancias et al., 2014; Dowdle et al., 2014). Besides this, SARS-CoV-2 infection activates the hepcidin pathway, which, in turn, suppresses the exportation of Fe^2+^, leading to the progression of ferroptosis (Banchini et al., 2020).

The involvement of the system Xc^-^/GSH/GPX4 axis, another key pathway that regulates ferroptosis (Yang et al., 2014), has also been investigated. The expression of GPX4, a vital regulator of ferroptosis, is decreased, and the surface expression of system Xc^-^ in the hepatocytes of COVID-19 patients is lower than that in healthy individuals (Krishnan et al., 2021). Moreover, numerous investigations have demonstrated GSH deficiency in severe COVID-19 patients (Bartolini et al., 2021; Kumar et al., 2021). GSH supplementation has been proposed as adjunctive therapy for COVID-19 (Guloyan et al., 2020; Silvagno et al., 2020).

Like the iron and amino acid metabolic pathways, the lipid metabolic pathway also plays an essential role in the regulation of ferroptosis. During ferroptosis, PUFAs are first converted to PUFA–CoAs by acyl–CoA synthetase long-chain family member 4 (Kuch et al., 2014; Doll et al., 2017). The PUFA–CoAs are then esterified by lysophosphatidylcholine acyltransferase 3 to generate phospholipids containing polyunsaturated fatty acid chains (PUFA-PLs). Finally, lipoxygenases oxidized PUFA-PLs to generate excessive lipid ROS (Yang et al., 2016; Kagan et al., 2017). Some researchers have suggested that lipid peroxidation is a hallmark of poor outcomes in COVID-19 patients (Martin-Fernandez et al., 2021; Zarkovic et al., 2021).

Taken together, three major pathways of ferroptosis are involved in COVID-19, and along with the direct evidence of the SARS-CoV-2 infection-induced signature of ferroptosis, it is reasonable to deduce that there is an association between ferroptosis and COVID-19.

Next, we discuss the potential role of ferroptosis in SARS-CoV-2 infection-caused liver injury.

## Ferroptosis possibly participates in COVID-19-associated liver injury

**Figure 1:**
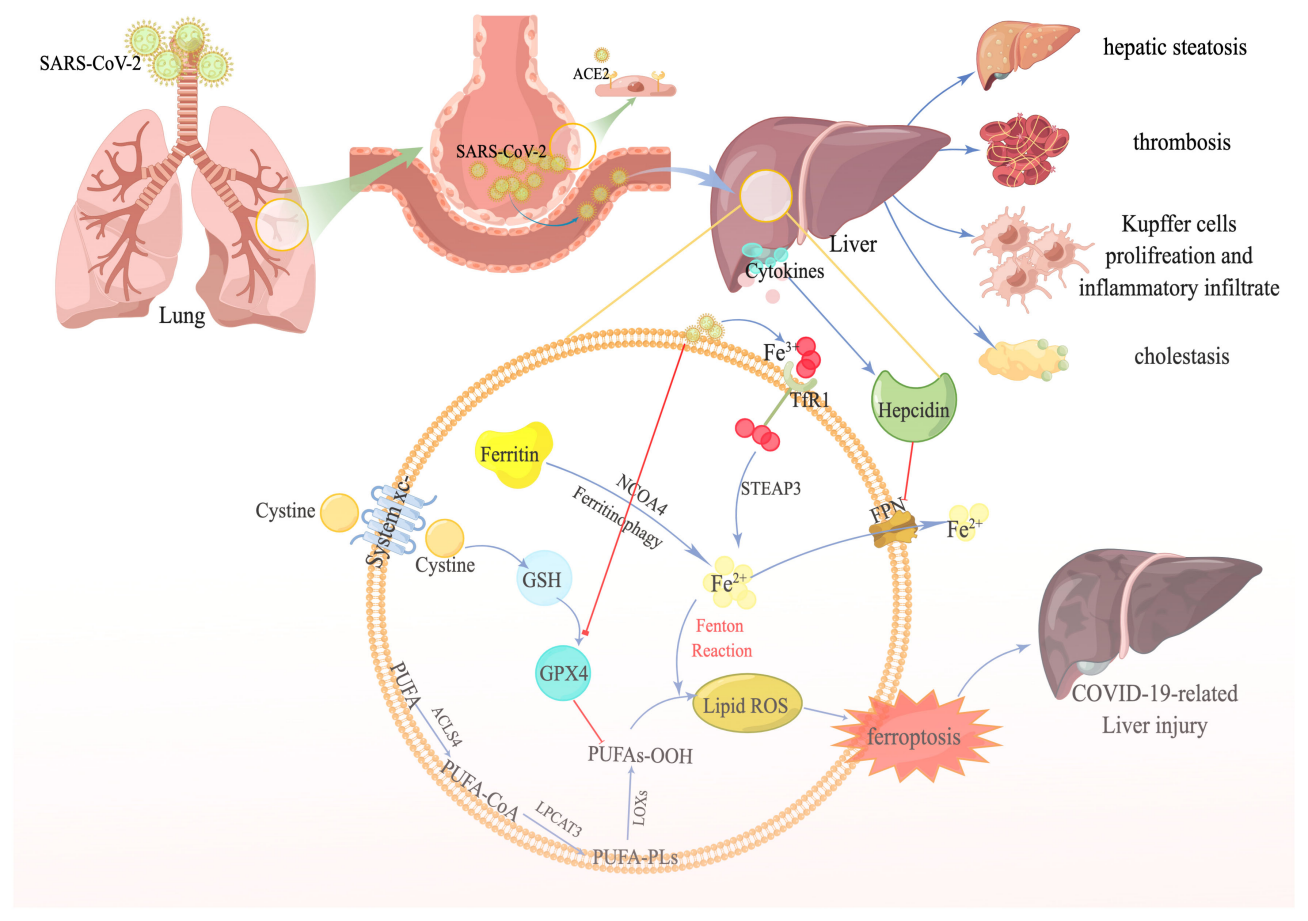
The potential link between ferroptosis and COVID-19-related liver injury. SARS-CoV-2 recognizes the angiotensin-converting enzyme 2 receptor in the alveoli, especially type II alveolar cells. SARS-CoV-2 subsequently reaches the blood circulation through the damaged blood–air barrier. Next, SARS-CoV-2 further infects the liver. After the infection, a plethora of transferrins carrying Fe^3+^ is transferred into the cell through transferrin receptor1. Eventually, Fe^3+^ is transformed to Fe^2+^ by STEAP family member 3, in addition to the Fe^2+^ degraded from excessive ferritin by nuclear receptor coactivator 4—contributing to the accumulation of ferrous iron in the cell. Numerous cytokines are also released during the infection, stimulating hepcidin expression, suppressing ferroportin, and aggravating the accumulation of iron even more. On the other hand, SARS-CoV-2 decreases the expression of GPX4, facilitating the iron overload-induced Fenton reaction, accompanied by polyunsaturated fatty acids, and producing massive amounts of lipid reactive oxygen species. Ultimately, ferroptosis occurs and causes liver injury.

Although the molecular mechanisms by which ferroptosis causes liver diseases are largely unknown, mounting evidence indicates that ferroptosis is fundamental to the pathogenesis of numerous types of liver diseases (Chen et al., 2022)—for example, ferroptosis probably promotes the progression of NASH by activating inflammatory responses, oxidative stress, and cell damage (Tsurusaki et al., 2019). In addition, ferroptosis is associated with the replication of hepatitis C virus (Yamane et al., 2021) and liver fibrosis (Yuan et al., 2022). Therefore, we hypothesize that ferroptosis might be involved in COVID-19-related liver injury as well (**Figure 1**).

Notably, the histopathological changes in the livers of patients with SARS-CoV-2 infection largely consist of hepatic steatosis, Kupffer cell activation, thrombosis, and inflammatory infiltration (Sonzogni et al., 2020; Diaz et al., 2020; Lagana et al., 2020; Zhao et al., 2021). Here we examine the potential links between ferroptosis and these pathological features.

### Ferroptosis and hepatic steatosis

Hepatic steatosis is defined as increased lipid accumulation in hepatocytes of at least 5% of liver weight, usually caused by the disruption of hepatic lipid homeostasis (Manne et al., 2018). A postmortem study of 48 patients who died from COVID-19 in two main hospitals in northern Italy noted hepatic steatosis in 54% of the samples (Sonzogni et al., 2020). Besides this, a meta-analysis of autopsy data from five studies, including 116 COVID-19 patients, also observed that more than half of the patients infected with SARS-CoV-2 displayed hepatic steatosis (Diaz et al., 2020). In addition to autopsy studies, the computed tomography scans of SARS-CoV-2-positive patients have exhibited higher frequencies of hepatic steatosis as well (Medeiros et al., 2020).

In patients with NASH, hepatocytic ferroptosis is arguably the trigger for inflammation (Tsurusaki et al., 2019), and liproxstatin-1 could reverse the severity of hepatic steatosis in mice fed with a methionine/choline-deficient diet (Qi et al., 2020). Additionally, enoyl coenzyme A hydratase 1, a key component in mitochondrial fatty acid β-oxidation, alleviates hepatic steatosis by inhibiting ferroptosis (Liu et al., 2021). Hence, suppressing ferroptosis could be a therapeutic strategy for ameliorating COVID-19-caused hepatic steatosis.

### Ferroptosis and thrombosis

There is evidence that SARS-CoV-2 stimulates thrombosis as a result of increased coagulation and decreased fibrinolysis (Sastry et al., 2022). The SARS-CoV-2 virus enters the endotheliocyte through the ACE2 receptor, leading to the accumulation of inflammatory cells and release of cytokines. As a result, endothelial cell death and vessel wall injury occur and finally contribute to the formation of thrombus (Shao et al., 2021). Nuclear factor erythroid 2-related factor 2 constitutes an imperative target for the suppression of ferroptosis and associated endothelial cell inflammation and thrombosis (Shao et al., 2021). Furthermore, ferroptosis and platelet activation are interconnected, accompanied by proteasome and inflammasome activations, leading to thromboembolism (NaveenKumar et al., 2019).

### Ferroptosis and inflammatory infiltration

Intense inflammatory reactions have been recorded in the portal and lobular regions during a SARS-CoV-2 infection. Meanwhile, Kupffer cells, an indispensable component of the liver, have also been shown to be activated and to participate in the inflammatory process (Nardo et al., 2021). In fact, ferroptosis and inflammatory infiltration in COVID-19 might be of reciprocal causation, forming a vicious circle. In the course of COVID-19, plenty of cytokines are released, among which interleukin-6 (IL-6) is the precise factor that stimulates hepcidin and ferritin syntheses (Moore and June, 2020). Eventually, the increments in hepcidin and ferritin contribute to iron overload, Kupffer cell activation (Li et al., 2022), and ferroptosis. Once the ferroptosis of hepatocytes occurs, damage-associated molecular patterns, inflammatory cytokines, and chemokines are released (Martin-Sanchez et al., 2017), which, in turn, enhance the infiltration of inflammatory cells and further aggravate COVID-19-associated liver injury.

Taken together, despite no direct evidence yet demonstrating the role of ferroptosis in SARS-CoV-2 infection-related liver injury, we believe that ferroptosis participates in the process of COVID-19-related liver injury in the following ways: (1) ferroptosis is closely associated with hepatic steatosis, which is one of the main characteristics of COVID-19-related liver injury; (2) SARS-CoV-2 stimulates the release of hepcidin, a peptide hormone produced by hepatocytes, leading to iron overload in the liver and triggering ferroptosis; and (3) SARS-CoV-2 infection disrupts the metabolism of lipids, potentially promoting lipid peroxidation in the context of iron overload. Moreover, ferroptosis-related liver injury in the course of a COVID-19 infection may share some similar mechanisms with other liver diseases, including but not limited to inflammatory reactions, immune responses, oxidative stress, and cell damage. Naturally, because direct cytotoxicity resulting from viral replication is considered the main mechanism of SARS-CoV-2-induced hepatic injury, the impact of ferroptosis on the replication of the virus could be the major difference in ferroptosis-induced liver injury between COVID-19 and other non-viral liver diseases.

## Therapeutic opportunities

The discussion up to this point suggests a close link between ferroptosis and COVID-19-associated liver injury. Hence, targeting ferroptosis could be a promising therapeutic option. So far, ferroptosis can be suppressed mainly by iron chelators and lipophilic antioxidants. Iron chelators, like DFO, deferasirox, and deferiprone, chelate iron and prevent lipid peroxidation by regulating the Fenton reaction. Lipophilic antioxidants, including ferrostatin-1 (Fer-1), liproxstatin-1 (Lip-1), and α-tocopherol, scavenge lipid peroxides and block ferroptosis.

Deferoxamine has been shown to inhibit the replication of human immunodeficiency virus type-1 (Georgiou et al., 2000) and enhance hepatitis B virus infection response to IFN-α treatment (Bayraktar et al., 1996). In addition, deferoxamine decreases the levels of IL-6, the central inflammatory cytokine released during COVID-19, indicating that deferoxamine could be a potential drug treatment for COVID-19-induced liver injury. However, others have argued that deferoxamine is probably harmful to COVID-19 since iron chelators aggravate anemia from inflammation, weakening the ability of the host innate immunity to be counterproductive (Garrick and Ghio, 2021).

Fer-1 and Lip-1 are well-known ferroptosis inhibitors. Numerous studies have demonstrated that these inhibitors prevent the progression of liver steatosis and fibrosis (Qi et al., 2020; Liu et al., 2021; Zhu et al., 2021); however, no investigation has yet assessed the impact of their efficiency in the treatment of COVID-19. α-Tocopherol is a type of vitamin E reported to have the ability to suppress the SARS-CoV-2-RNA-dependent RNA polymerase (Pacl et al., 2021) and inhibit the main protease (Linani et al., 2022). Selenium, n-acetylcysteine, and polyphenols are all antioxidants and are regarded as ferroptosis suppressors; however, the administration of n-acetylcysteine yielded no benefit to severe COVID-19 sufferers, and the effects of selenium and polyphenols on COVID-19 are yet to be determined in larger clinical trials (Iddir et al., 2020; de Alencar et al., 2021; Balboni et al., 2022).

Notably, SARS-CoV-2 infection-induced tissue injury-related cell death is not limited to ferroptosis. Several other types of regulated cell deaths have also been linked to COVID-19 (Yapasert et al., 2021; Paolini et al., 2021). Therefore, simultaneously inhibiting different cell death types could alleviate SARS-CoV-2-induced injuries. Necrostatin-1, a widely used inhibitor of necroptosis, suppresses ferroptosis as well (Tonnus et al., 2021). It has been shown to protect against acetaminophen-induced hepatotoxicity (Takemoto et al., 2014; Saleh et al., 2021), which possibly participates in drug-generated liver injury during COVID-19.

Furthermore, inflammatory cytokine storms are thought to be critical factors in the causation of multiple organ failure syndrome in severe COVID-19 patients. Combining anti-inflammatory cytokines and interfering with ferric ion metabolism to improve the patients’ resilience would be promising and should be investigated in future studies.

## Conclusion and perspectives

Liver injury is common in patients infected with SARS-CoV-2, but the mechanism of COVID-19-related liver injury is largely unknown. Given the potential association between ferroptosis and liver histopathological changes in patients with COVID-19, we hypothesize that ferroptosis participates in SARS-CoV-2 infection-related liver injury. Targeting ferroptosis could be a promising strategy to reduce COVID-19-related hepatic injury.

However, to the best of our knowledge, there is no evidence of ferroptosis signature in the liver tissues of COVID-19 patients, let alone clinical trials evaluating inhibitors of ferroptosis in COVID-19-related liver injury. Although much progress has been made in our understanding of the pathological role of ferroptosis in liver diseases, we still have no idea of the precise role of ferroptosis, if any, in SARS-CoV-2-impaired liver and how ferroptosis drives the initiation of inflammation contributing to liver injury. By extension, we even have no idea whether ferroptosis during COVID-19 is the result of SARS-CoV-2 infection or if it is a compensatory mechanism to facilitate the replication and toxicity of SARS-CoV-2. Generally, several mechanisms are involved in ferroptosis, and we have described the link between three major ferroptosis pathways and COVID-19. However, we have no idea whether all these elements play significant roles in COVID-19-related hepatic injury.

Recent studies have identified apoptosis (Wang et al., 2020; Shirazi Tehrani et al., 2022) and autophagy (Shirazi Tehrani et al., 2022), the two most widely known RCD types, in the hepatic tissues of patients with COVID-19 as well. Moreover, pyroptosis, another form of RCD, is also said to contribute to COVID-19 pathogenesis (Junqueira et al., 2022). Therefore, it is rather difficult to decipher which RCD type is dominant in COVID-19-related liver injury. Studies using different antagonists to target corresponding cell death types in SARS-CoV-2-caused liver injury models could provide a better understanding of the matter.

Furthermore, even if ferroptosis can be considered a therapeutic target for COVID-19-related hepatic injury, other questions must still be answered: which sensitive biomarkers of ferroptosis can be identified, when should therapy start, and how can ferroptosis be inhibited without affecting healthy cells? What is more, the use of iron chelators could impact the hepatic iron metabolism, which, in turn, would disrupt iron-related cellular processes, such as energy production, oxygen transport, and DNA synthesis (Sheftel et al., 2012; Dev and Babitt, 2017) as well as impair immune response to the pathogen (Girelli et al., 2021). All these questions must be resolved to provide a complete and convincing argument for the clinical application of ferroptosis inhibitors.

Cell death is the termination of various pathophysiological processes and the basis of tissue injury. As a novel but important cell death type, ferroptosis might be a new and effective target for preventing and treating hepatic damage after a SARS-CoV-2 infection. Investigations are needed to establish the occurrence of ferroptosis in COVID-19 and elucidate its exact mechanism as well as its association with COVID-19-related liver injury.

## Author Contributions

LS and YX drafted the manuscript, KZ and MD edited the manuscript, and YC designed the study and revised the manuscript. All authors contributed to the article and approved the submitted version.

## Funding

This research was funded by the Special Anti-epidemic Project by hospitals directly affiliated with universities—A Project Supported by the Scientific Research Fund of Zhejiang Provincial Education Department (grant no. Y202043882), Jiaxing Key Laboratory of Virus-Mediated Infectious Diseases (grant no. 2021-bdzdsys), Jiaxing Research Institute of Hepatology (grant no. jxsgbyjs), Jiaxing Key Supporting Discipline of Medicine (grant no. 2019-zc-02), and Program of the First Hospital of Jiaxing (grant no. 2021-YA-001).

## Acknowledgments

We thank Figdraw (www.figdraw.com) for helping us produce the figures.

## Conflict of Interest

The authors declare that the research was conducted in the absence of any commercial or financial relationships that could be construed as a potential conflict of interest.

## Publisher’s Note

All claims expressed in this article are solely those of the authors and do not necessarily represent those of their affiliated organizations, or those of the publisher, the editors and the reviewers. Any product that may be evaluated in this article, or claim that may be made by its manufacturer, is not guaranteed or endorsed by the publisher.
